# Integrating Deep Learning and Radiogenomics: A Novel Approach to Glioblastoma Segmentation and MGMT Methylation Prediction

**DOI:** 10.3390/jimaging11110403

**Published:** 2025-11-11

**Authors:** Nabil M. Abdelaziz, Emad Abdel-Aziz Dawood, Alshaimaa A. Tantawy

**Affiliations:** Information Systems Department, Faculty of Computers and Informatics, Zagazig University, Zagazig 44519, Egypt; nmabedelaziz@fci.zu.edu.eg (N.M.A.); eatantawi@fci.zu.edu.eg (A.A.T.)

**Keywords:** glioblastoma, brain tumors, hybrid model, MRI images, U-Net architecture, MGMT methylation status

## Abstract

Radiogenomics, which integrates imaging phenotypes with genomic profiles, enhances diagnosis, prognosis, and treatment planning for glioblastomas. This study specifically establishes a correlation between radiomic features and MGMT promoter methylation status, advancing towards a non-invasive, integrated diagnostic paradigm. Conventional genetic analysis requires invasive biopsies, which cause delays in obtaining results and necessitate further surgeries. Our methodology is twofold: First, an enhanced U-Net model segments brain tumor regions with high precision (Dice coefficient: 0.889). Second, a hybrid classifier, leveraging the complementary features of EfficientNetB0 and ResNet50, predicts MGMT promoter methylation status from the segmented volumes. The proposed framework demonstrated superior performance in predicting MGMT promoter methylation status in glioblastoma patients compared to conventional methods, achieving a classification accuracy of 95% and an AUC of 0.96. These results underscore the model’s potential to enhance patient stratification and guide treatment selection. The accurate prediction of MGMT promoter methylation status via non-invasive imaging provides a reliable criterion for anticipating patient responsiveness to alkylating chemotherapy. This capability equips clinicians with a tool to inform personalized treatment strategies, optimizing therapeutic efficacy from the outset.

## 1. Introduction

Brain tumors are among the most lethal conditions globally, contributing significantly to mortality in both children and adults. They result from the uncontrolled proliferation of cells, leading to the formation of abnormal masses in the brain [1]. These tumors are generally categorized as primary or secondary, classified by their growth rate and origin. Primary brain tumors are particularly notorious, as some types demonstrate rapid and aggressive growth, making them exceedingly difficult to treat.

Glioma is a type of primary brain tumor. Gliomas represent approximately 30% of all primary brain and central nervous system tumors, and notably, about 80% of all malignant brain tumors are gliomas, with glioblastoma being the most common and aggressive subtype. Gliomas are further classified into different grades based on their severity and growth rates. Grades II and III gliomas, for instance, are known for their rapid progression, necessitating urgent medical intervention [2]. Glioblastomas are notorious for their fast growth, resistance to treatment, and poor prognosis, making them one of the most formidable challenges in oncology. Despite intensive research, the prognosis for glioblastoma patients remains poor, with a median survival of approximately 15 months. While incremental advances, such as the introduction of tumor-treating fields (TTFields), have been made, they have resulted in only modest extensions in progression-free and overall survival for specific patient subgroups, underscoring the urgent need for more effective strategies. Most patients still face tumor recurrence and have a poor prognosis [3].

Glioblastomas are also characterized by their extreme variability in form and microscopic structure, particularly in adult cases where they are classified as diffuse astrocytic gliomas. These tumors are considered the most lethal among central nervous system malignancies due to their complex nature [4].

The current classification of central nervous system (CNS) cancers, as defined by the World Health Organization, mandates an integrated diagnostic model that incorporates key molecular and cytogenetic markers alongside traditional histology [5]. The integration of this multidimensional data is indispensable for deciphering tumor heterogeneity, thereby increasing diagnostic precision and paving the way for more effective, patient-specific treatment plans [6].

In recent years, the application of artificial intelligence and deep learning has shown great promise in the area of diagnostic medicine, particularly in the analysis and interpretation of complex datasets. Deep learning involves training models to recognize intricate patterns in various forms of data, including text, images, and audio. These models mimic the learning processes of the human brain, allowing them to generate accurate insights and predictions that can significantly aid in medical decision-making. Deep learning’s potential to revolutionize the diagnosis and treatment of brain tumors, particularly gliomas, resides in its capacity to swiftly and precisely manage and analyze enormous volumes of data. This technological advancement offers hope for the development of more effective diagnostic tools and treatment strategies, improving the prognosis for patients suffering from these devastating conditions.

### 1.1. (MGMT) O6-Methylguanine-DNA Methyltransferase Enzyme

The MGMT (O6-methylguanine-DNA methyltransferase) gene encodes a DNA repair enzyme that confers resistance to alkylating chemotherapy agents, such as temozolomide, by reversing DNA damage at guanine nucleotides [7]. However, methylation of the MGMT promoter silences its expression, leading to a loss of this DNA repair mechanism [8]. Consequently, glioblastoma patients with a methylated MGMT promoter are more responsive to alkylating chemotherapy, resulting in significantly better treatment outcomes and survival [9]. In contrast, an unmethylated promoter allows for functional MGMT protein production, enabling tumor cells to repair chemotherapeutic damage and leading to inherent treatment resistance and poorer prognosis.

### 1.2. U-Net Architecture

For brain tumor segmentation, we employed the U-Net architecture [10], which is particularly well-suited for biomedical image analysis due to its encoder–decoder structure with skip connections. These connections are crucial for preserving fine-grained spatial details during upsampling, enabling precise localization of irregular and diffuse glioblastoma tumor boundaries. Consequently, it generates more precise segmentation masks, as these skip connections enhance the network’s ability to comprehend the relationships between various image segments, leading to improved segmentation results [11]. Magnetic Resonance Imaging (MRI) is the standard medical technology used for classifying and segmenting various tissue abnormalities in the brain, particularly brain tumors.

Numerous methodologies have been proposed for this purpose. However, traditional approaches face several challenges:

The segmentation and classification of brain tumors involve processing a vast amount of data, which can be quite complex.

Factors such as limited acquisition time and the indistinct boundaries of soft tissues can significantly impact the accuracy of these processes.

The irregular shapes and sizes of tumors further complicate the effectiveness of existing methods.

These challenges highlight the need for a novel approach to improve the classification and segmentation of brain tumors.

This paper introduces a hybrid deep learning model for the dual task of brain tumor tissue segmentation and MGMT promoter methylation status classification. The architecture leverages a U-Net foundation for segmentation, augmented by parallel feature extraction streams from pre-trained EfficientNetB0 and ResNet50 models. This design synergistically combines EfficientNetB0’s computational efficiency in multi-scale feature extraction with ResNet50’s strengths in deep, hierarchical pattern recognition. The fusion of their feature maps enriches the model’s representational capacity, aiming to achieve superior accuracy, robustness, and generalizability in analyzing complex neuro-oncological imaging data.

The clinical significance of this model lies in its potential to non-invasively predict MGMT promoter methylation status. Accurate pre-operative determination of MGMT status is a critical prognostic and predictive biomarker, as methylation sensitizes glioblastomas to temozolomide (TMZ) chemotherapy [12]. Conventional methods for assessing MGMT status rely on surgical tissue biopsies, which are invasive and carry inherent risks, including infection and sampling bias. By providing a reliable, imaging-based alternative, our model could empower clinicians to personalize neoadjuvant or adjuvant treatment strategies earlier in the clinical pathway. This paradigm shift towards non-invasive genotyping holds the potential to optimize therapeutic efficacy, spare patients from ineffective treatments and their associated toxicities, and ultimately improve overall survival outcomes.

## 2. Literature Survey

Jingyu Zhu et al. [13] (August 2025) introduced TAUM-Net, a multitask framework that jointly segments tumors and predicts MGMT methylation from MRI. The model fuses CNN modules for fine, local tumor features with a Transformer to capture global anatomical context and splits into two task-specific branches—one for refining segmentation masks and another for aggregating multi-scale cues for methylation classification—while sharing a common learned representation. On BraTS2021 and TCGA-GBM, TAUM-Net achieves a Dice score of 0.921 for segmentation and 63.48% accuracy for MGMT prediction, showing that multitask learning can preserve strong segmentation performance and offer reasonable predictive power for molecular status.

Sumaiya Fazal (March 2025) introduced ADAPT (Adaptive Sparse Autoencoders), an innovative method for determining MGMT methylation status in glioblastoma patients using MRI images [14]. ADAPT offers a promising advancement for personalized treatment plans in glioblastoma by improving the accuracy of MGMT methylation status prediction. Its high performance in terms of accuracy, specificity, and sensitivity suggests strong potential for clinical application, enhancing both diagnosis and treatment response predictions for glioblastoma patients. The ADAPT method achieves 95% accuracy, 93% specificity, and 94% sensitivity. These results highlight the method’s high diagnostic precision.

İlker Özgür Koska et al. (January 2025) aimed to develop a robust classifier for predicting MGMT methylation status in glioblastoma using multi-parametric MRI [15]. By utilizing a subset of the BRATS 2021 dataset with MGMT labels and segmentation masks, researchers created a comprehensive mask fusion approach that identified relevant tissue areas, including those that appear disease-free yet contain pathology. They built a 3D ROI-based custom CNN classifier, which outperformed single-sequence classifiers, achieving accuracies of 0.88 with a multi-parametric approach using T1 contrast-enhanced and FLAIR images and 0.81 with all four MRI sequences. The best model also achieved an ROC AUC of 0.90.

Pavan Nathani et al. (November 2024) presented a radiogenomic model for predicting Methylguanine DNA Methyltransferase (MGMT) promoter hypermethylation in Glioblastoma Multiforme (GBM) using multimodal MRI data and the EfficientNet deep learning architecture [16]. Since MGMT status is critical for determining temozolomide sensitivity and improving prognosis, a non-invasive prediction method is essential to reduce the need for biopsies. The study utilized MRI sequences from the Brain Tumor Segmentation (BraTS) 21 dataset, including T1-weighted, T1-weighted contrast-enhanced, T2-weighted, and FLAIR scans, focusing on preprocessing techniques for cross-modal alignment. The EfficientNet-B0 model, pre-trained on ImageNet and fine-tuned for binary classification of MGMT methylation status, achieved a moderate validation score of 0.62393.

Shaz Mumtaz Khan et al. (September 2024) presented an effective and innovative approach to brain tumor detection and classification, integrating deep learning with advanced feature selection methods to improve precision and efficiency in medical imaging [17]. Geometric features and four texture attributes were extracted and optimized through a step-by-step process using a Genetic Algorithm (GA). The proposed method achieved a high accuracy of 96% in identifying and classifying brain tumors. The model’s performance and novelty were rigorously compared with established techniques, demonstrating superior results.

Seong-O Shim (March 2024) applied the ResNet101 deep learning model with transfer learning for analyzing the 2021 Radiological Society of North America (RSNA) Brain Tumor Challenge dataset [18]. For determining MGMT methylation state in glioma patients, the model achieved an accuracy of 85.48%, sensitivity of 80.64%, and specificity of 90.32%. Conversely, for classifying cases with no tumor, the model yielded an accuracy of 85.48%, sensitivity of 90.32%, and specificity of 80.64%. Additionally, 74 radiomic features were computed and optimized using an ensemble Bagged Tree classifier combined with the Relief feature selection method, which reduced the feature set to 30. This enhanced approach improved validation accuracy to 84.3% and gave a result for the area under the curve (AUC) equal to 0.9038 for detecting MGMT promoter methylation status.

Yongqi He et al. (February 2024) focused on predicting the methylation status of the MGMT (O-6-methylguanine-DNA methyltransferase) promoter in glioblastoma using weakly supervised learning and transformer-based models [19]. The methylation status of MGMT is important for treatment and prognosis, but its detection remains challenging. The authors employed a weakly supervised learning approach, which allows the model to learn from the data with limited labeling. This was achieved using two transformer-based models, which are known for their effectiveness in handling complex image data. Accuracy and AUC (Area Under the Curve) scores were used to evaluate model performance. TCGA dataset: accuracy = 0.79, AUC = 0.86. Independent dataset (Beijing Tiantan Hospital): Accuracy = 0.76, AUC = 0.83.

Shenbagarajan et al. (February 2024) presented a method to enhance brain tumor detection in MRI scans using advanced machine learning and deep learning techniques [20]. Their approach preprocesses MRI images with the Adaptive Contrast Enhancement Algorithm and median filtering. The classification of tumor regions is performed using a novel Ensemble Deep Neural Support Vector Machine (EDN-SVM) classifier. The model achieved outstanding performance, with 97.93% accuracy, 92% sensitivity, and 98% specificity, highlighting its potential as a reliable tool for accurately detecting abnormal and normal brain tissues in MRI scans.

## 3. Data Source and Collection

**Figure 1:**
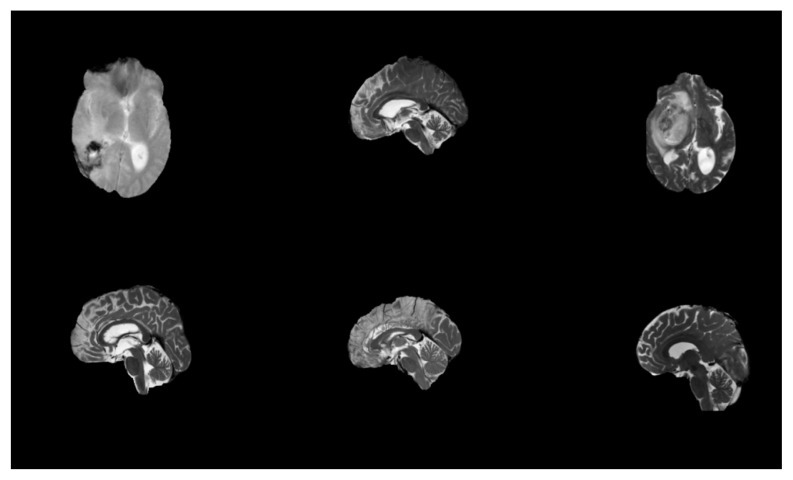
Original MRI scans from the dataset.

The dataset BraTS-2021 (Brain Tumor Segmentation 2021) is a benchmark dataset commonly applied in the areas of medical scan analysis, specifically for the study of brain tumor segmentation. It is part of an ongoing challenge aimed at improving the automatic brain tumor subregion segmentation from multimodal MRI images. mpMRI images are instrumental for researchers developing algorithms for automatic segmentation and classification of brain tumors, pushing forward the capabilities in radiogenomic and personalized treatment plans. The BraTS 2021 dataset used in this study comprises multi-institutional pre-operative mpMRI scans from a total of 2041 subjects. For the specific task of MGMT promoter methylation prediction, we utilized the subset provided for the RSNA-MICCAI BraTS 2021 Challenge, which includes 585 subjects with known MGMT labels (Training set). Each subject contains four aligned MRI sequences (T1, T1-CE, T2, FLAIR) in the form of 3D NIfTI volumes. The typical matrix size is 240 × 240 × 155 voxels per modality. The dataset was accessed via Kaggle and required approximately 7 GB of storage. In May 2021, the dataset was available on the Kaggle website. The composition of these three cohorts is as follows: A five-digit number designates the specific folder for each case. The dataset’s mpMRI images are displayed in Figure 1. The BraTS-2021 dataset features multimodal MRI (mpMRI) images, which are crucial for effective brain tumor analysis and segmentation, as stated in reference [21]. Specifically, the dataset includes four key MRI modalities: T1-weighted MRI: This provides detailed anatomical information and is useful for assessing the overall brain structure. However, it may not clearly differentiate between tumor components. T1-weighted contrast-enhanced (T1-CE) MRI: This modality uses a contrast agent to highlight areas with disrupted blood–brain barriers, typically indicating the presence of an enhancing tumor. It is particularly effective for visualizing the tumor’s active regions. T2-weighted MRI: This modality offers excellent contrast for identifying edema surrounding the tumor. It helps in distinguishing the tumor from surrounding tissues, highlighting areas of water content, which can be indicative of tumor mass. Fluid-Attenuated Inversion Recovery (FLAIR) MRI images are particularly useful for identifying abnormalities that might be less visible in other modalities. The combination of these modalities allows for a more comprehensive analysis of brain tumors by providing several types of information about the tumor and surrounding brain tissues. The two main objectives of the suggested method are the segmentation of the tumor subregion and the radiogenomic categorization of the methylation status of the MGMT promoter.

**Figure 2:**
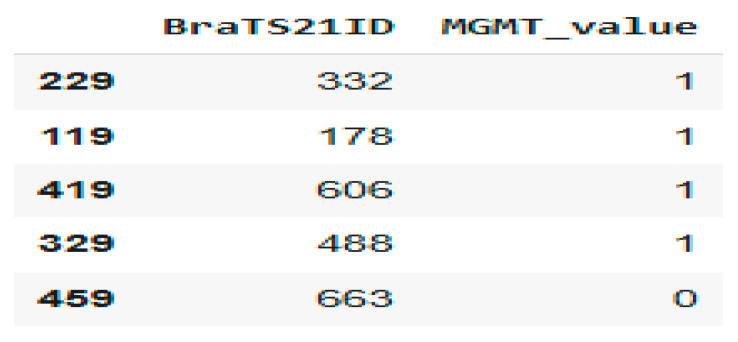
Patient MGMT Status Overview from BraTS21 Dataset.

Figure 2 shows a sample from the dataset related to the MGMT methylation status for brain tumors. Here is a brief interpretation of the data.

BraTS21ID: This is an identifier for brain tumor cases in a dataset from the BraTS (Brain Tumor Segmentation) challenge. MGMT_value: This indicates the MGMT promoter methylation status: 0: Not methylated; 1: Methylated.

## 4. Materials and Methods

**Figure 3:**
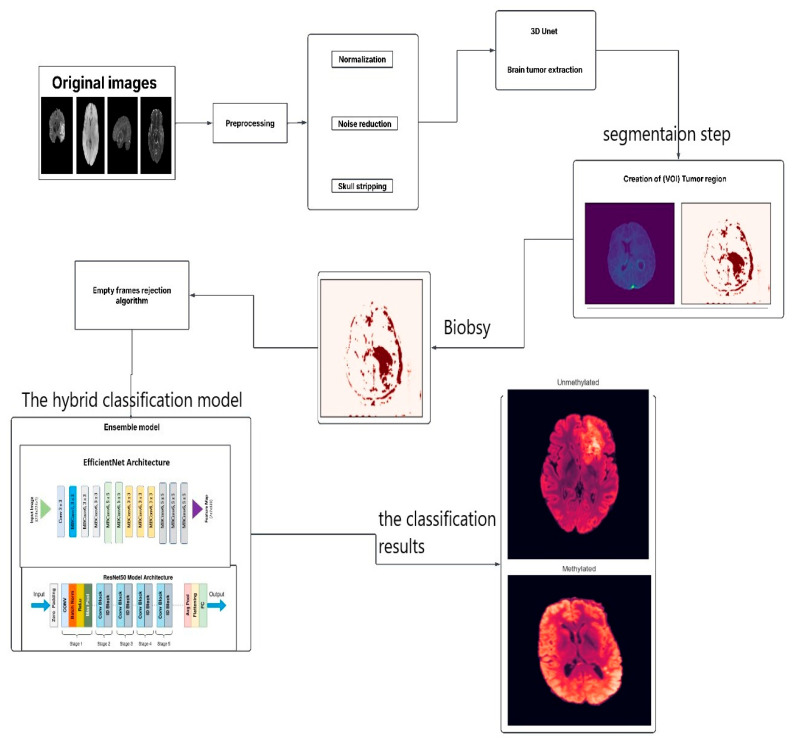
The Proposed Model.

This study proposes a hybrid deep learning model that integrates medical imaging with genetic analysis to enhance the diagnostic characterization of glioblastoma. The overarching pipeline, illustrated in Figure 3, follows a structured workflow of data acquisition, preprocessing, feature extraction, model training, and validation. Following careful curation and preprocessing of mpMRI data to ensure quality and consistency, the model extracts discriminative features to predict the critical genetic biomarker, MGMT promoter methylation status.

Architecture employs a hybrid approach by integrating two pre-trained convolutional neural networks (CNNs). This design leverages the complementary strengths of EfficientNetB0, renowned for its parameter efficiency and powerful feature extraction capabilities, and ResNet50, which excels at learning complex hierarchical patterns through its deep residual connections. By fusing the feature maps from these networks, the model achieves a rich and multi-scale representation of tumor phenotypes, enabling accurate non-invasive genotyping to inform therapy planning.

The data preparation pipeline for model training commenced with the loading of ground-truth labels from the train_labels.csv file, which annotates each case for tumor presence (1) or absence (0). Subsequently, the corresponding preprocessed and segmented magnetic resonance imaging (MRI) scans were retrieved. These scans were then converted into a standardized tensor format suitable for deep learning model input. To render the native 3D NIfTI volumes compatible with pre-trained 2D architectures, a standardized preprocessing pipeline was implemented. For each patient, 2D axial slices encompassing the tumor were extracted from three key modalities: T1-weighted contrast-enhanced (T1-CE), T2-weighted (T2), and FLAIR. Individual slices were resized to 256 × 256 pixels. Co-registered slices from the three sequences were then stacked to form a 3-channel input, analogous to an RGB image, thereby enabling the model to process multi-parametric information concurrently. Pixel values were normalized to a [0, 1] range.

The associated MGMT promoter methylation status (MGMT_value) was used as the label for supervised learning. The dataset was partitioned at the patient level into training (80%), validation (10%), and test (10%) sets. This stratification ensures that all data from a single patient is confined to one subset, preventing data leakage. The training set was used for model optimization, the validation set for hyperparameter tuning, and the held-out test set for the final evaluation of generalization performance on unseen data. This structured preparation establishes a robust foundation for model training and evaluation.

The implementation of this model involves several steps. The flowchart illustrates the process for analyzing brain MRI images to identify the presence and characteristics of brain tumors, specifically focusing on glioblastoma. Here is a breakdown of each step:

1. Original Images: The process begins with the acquisition of original MRI images of the brain.
2. Preprocessing: This crucial step includes normalization, adjusting the intensity values across images for consistency, and noise reduction by removing irrelevant noise from the images to improve clarity. Skull Stripping: Eliminating non-brain tissues from the images to focus on brain structures.
3. 3D U-Net: A specialized model is employed to extract brain tumors from preprocessed images.
4. Creation of VOI (Volume of Interest) Tumor Region: After extracting the tumor, a region of interest is defined, focusing on the areas affected by the tumor.
5. Biopsy: In this context, a biopsy is conducted to further evaluate the tumor.
6. Empty Frames Rejection Algorithm: This step filters out any images that do not contain relevant data or unreliable predictions, ensuring only meaningful frames are processed to enhance the robustness of the diagnostic framework.
7. Ensemble Model: This step integrates two deep learning architectures: EfficientNet Architecture, a model designed for efficient feature extraction from the images, and ResNet50 Model Architecture, a model capable of learning complex patterns.
8. Output: The last step categorizes the tumor status into:
  - Unmethylated: Indicating the MGMT promoter is not methylated.
  - Methylated: Indicating the MGMT promoter is methylated.

This comprehensive process employs advanced imaging and machine learning techniques to assist in accurately diagnosing and categorizing glioblastoma.

Once the model has been trained, its performance is assessed using a validation dataset. This evaluation will provide insights into the model’s accuracy, sensitivity, and specificity in predicting the MGMT methylation status based on the MRI data. Realizing the research objectives will allow brain tumor patients to benefit from less invasive diagnostic methods and treatments. Implementing personalized initial treatment strategies prior to surgery can enhance therapeutic efficacy and patient survival.

### 4.1. The Stage of Brain Tumor Extraction (Segmentation)

Tumor segmentation was performed using a U-Net deep learning architecture. The preprocessing pipeline involved resizing input images to the model’s required dimensions to generate a precise tumor region mask. The model’s output provides a pixel-wise classification that delineates the tumor from healthy brain tissue, with a specific focus on regions characteristic of glioblastoma.

**Figure 4:**
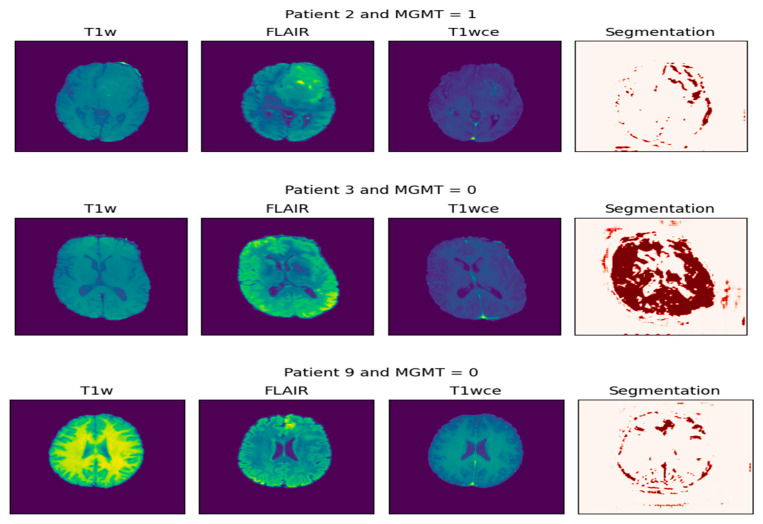
Extraction of tumor biopsy (the result of segmentation).

This process is visualized in Figure 4. The left panel displays the multi-parametric input, consisting of the original T1-weighted (T1W), FLAIR, and contrast-enhanced T1-weighted (T1wce) MRI sequences. The right panel presents the corresponding segmentation mask, where the tumor region is highlighted in red. This visualization effectively isolates the pathological area, providing a critical foundation for volumetric analysis, diagnostic confirmation, and subsequent treatment planning. Together, these panels demonstrate the initial step of non-invasively extracting a tumor’s spatial profile from multi-parametric MRI data.

**Figure 5:**
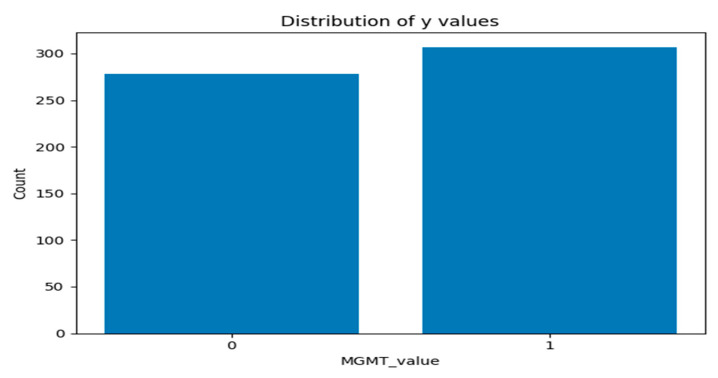
Graph of distribution of each MGMT_value.

**Figure 6:**
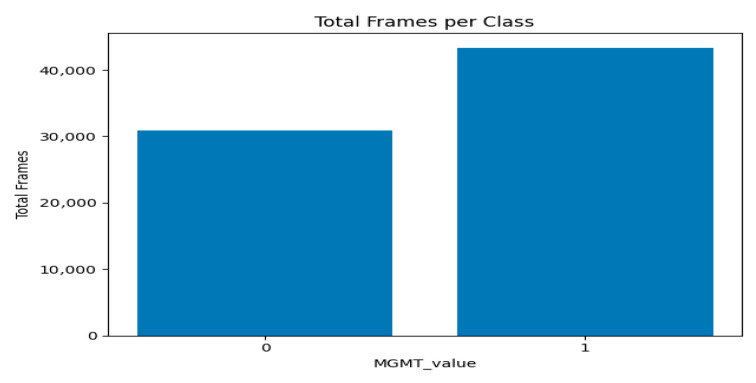
Graph of total number of image frames for each value per class.

Figure 5 and Figure 6 present two bar plots: one displays the distribution (count) of each MGMT_value, while the other shows the total number of image frames for each value. These visualizations help illustrate the class imbalance and data distribution, which are critical for developing machine learning models.

Class Distribution:

- Class 0 (Unmethylated MGMT promoter): 278 cases with 30,878 associated image frames.
- Class 1 (Methylated MGMT promoter): 307 cases with 43,370 associated image frames.

Following segmentation, a preprocessing algorithm was applied to exclude non-informative (empty or near-empty) axial slices from the dataset. This step enhances dataset quality by removing slices devoid of meaningful anatomical structure, thereby reducing computational load and potential noise during model training.

The algorithm operates on individual 2D slices, accepting both grayscale and multi-channel inputs. For each slice, the following procedure was implemented:

1. Grayscale Conversion and Normalization: Slices were converted to 8-bit grayscale, normalizing the dynamic range to [0, 255].
2. Foreground Mask Generation: A foreground mask was computed using Otsu’s thresholding, applied with both bright-on-dark and dark-on-bright polarities to account for varying intensity profiles.
3. Morphological Filtering: The resulting masks were refined using morphological opening and closing operations to remove small noise-induced artifacts. The polarity yielding the largest connected component was selected for subsequent analysis.
4. Quantification: The area of the largest connected component was measured for each slice and expressed both as an absolute pixel count and as a ratio of the total slice area.

A threshold was empirically determined from the distribution of area ratios to discriminate between informative and non-informative slices. In cases where clustering was degenerate, a conservative fallback utilizing a low quantile of the area ratios was employed. The efficacy of this filtering was validated by visually inspecting the separation between retained and excluded slices based on their area ratio distributions.

**Figure 7:**
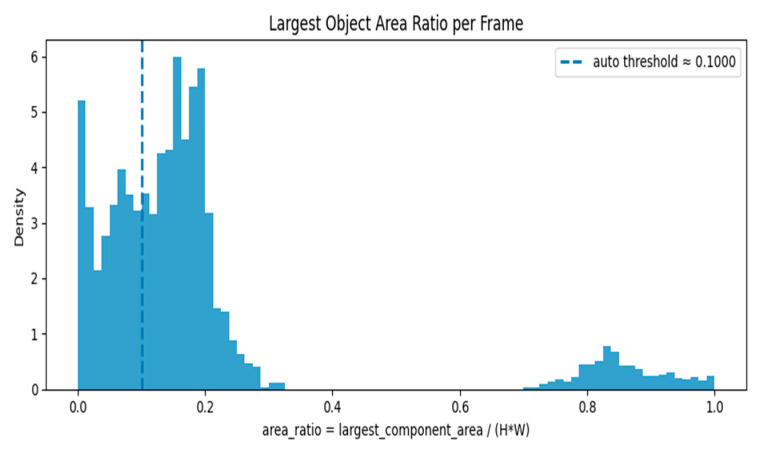
Largest Object Area Ratio per Frame—Histogram with Auto Threshold.

Figure 7 shows two major modes/clusters are visible:

A cluster concentrated near low area ratios (around 0.0–0.25): This cluster represents frames with small foreground objects or nearly empty/background frames.

A separate cluster near high area ratios (around 0.75–1.0): This cluster represents frames where a large portion of the image is occupied by foreground (e.g., strong anatomical content or large objects).

The auto threshold (~0.10) sits inside the low-ratio cluster, roughly separating very-small-object/empty frames (left of the line) from frames with moderate or larger objects (right of the line). The area ratio is calculated as largest_component_area/(H × W), where H and W are the frame height and width. The dashed line indicates the automatically calculated threshold of 0.1000. The histogram’s height (density) shows the relative frequency of frames at each area ratio value; the left peak implies many frames are nearly empty or have only tiny components.

**Figure 8:**
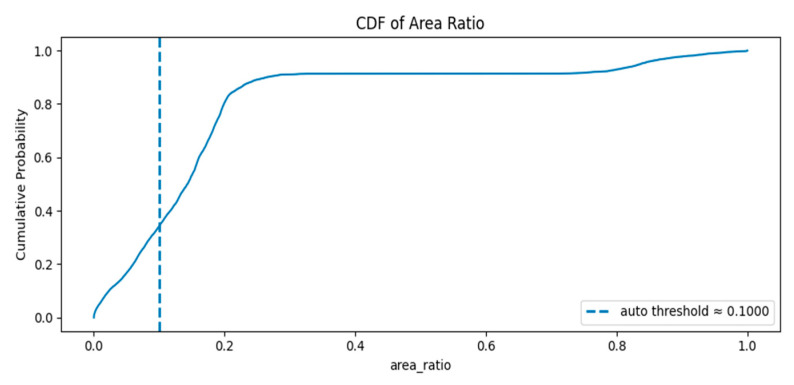
The cumulative distribution function (CDF) of per-frame area ratios.

Figure 8 displays the cumulative distribution function (CDF) of per-frame area ratios: Rapid rise around low ratios: The CDF climbs steeply between roughly 0.05 and 0.25, meaning a large proportion of frames have small to moderate area ratios.

Plateau near 0.9: By area ratio ≈ 0.25–0.4 the CDF flattens around ~0.9, indicating about 90% of frames have area ratios below that range.

Large-object tail: The remaining ~10% of frames occupy the high area ratio region (≈0.7–1.0), corresponding to frames where the foreground occupies most of the image.

The auto threshold (~0.10) lies in the rising portion of the CDF where the cumulative probability is between ~0.2 and ~0.4 (exact value visible on the plot). This means a non-trivial fraction of frames fall below the threshold and would be flagged as empty/minimal.

The classification model employs hybrid architecture that synergistically integrates two pre-trained convolutional neural networks (CNNs): EfficientNetB0 and ResNet50. This design leverages the complementary strengths of each network—EfficientNetB0’s parameter efficiency and powerful feature extraction capabilities, and ResNet50’s ability to learn complex hierarchical patterns through its residual connections, which mitigate the vanishing gradient problem in deep networks.

The architecture operates in three sequential phases:

1. Parallel Feature Extraction: The input image is processed simultaneously by both EfficientNetB0 and ResNet50. Each network extracts a distinct set of high-level feature maps, capturing diverse and complementary visual characteristics relevant to the pathology.
2. Feature Fusion: The resulting feature maps from both networks are concatenated into a unified, high-dimensional feature vector. This fusion enriches the representation capacity of the model by combining multi-scale and multi-abstraction-level information.
3. Classification Head: The fused feature vector is passed through a series of fully connected (dense) layers for final prediction. This segment comprises:
  ○ A dense layer with 512 units and ReLU activation.
  ○ A dropout layer (rate = 0.3) for regularization.
  ○ A second dense layer with 256 units and ReLU activation.
  ○ A subsequent dropout layer (rate = 0.3) to further enhance generalization.
  ○ A final output layer with a single neuron and a sigmoid activation function, which produces a probability score for the binary classification task. A score > 0.5 predicts a methylated MGMT promoter (Class 1), while a score ≤ 0.5 predicts an unmethylated status (Class 0).

**Figure 9:**
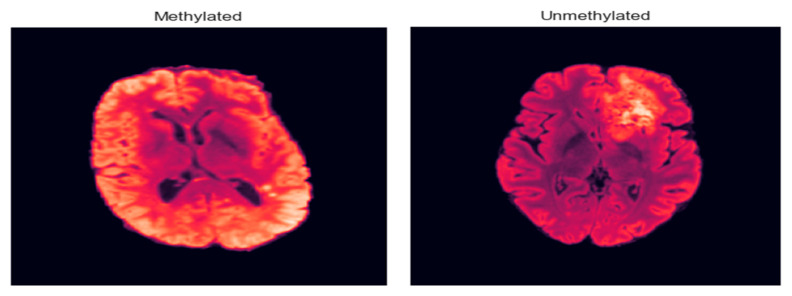
Methylated and unmethylated MGMT.

As illustrated in Figure 9, the model effectively distinguishes between representative MRI scans from unmethylated and methylated MGMT classes, demonstrating its capability to capture the imaging phenotypes associated with this critical genetic marker.

The hybrid model was compiled for the binary classification task using the binary cross-entropy loss function. Optimization was performed using the Adam optimizer with an initial learning rate of 0.001. To ensure a comprehensive evaluation, the model was configured to track accuracy, precision, recall, and F1-score throughout the training process.

The model was trained for 20 epochs using a mini-batch gradient descent approach with a batch size of 32. To enhance training efficiency and prevent overfitting, two key callbacks were implemented:

- Early Stopping: Training was halted if the validation accuracy failed to improve for five consecutive epochs, thereby preserving the model with the highest generalizability.
- ReduceLROnPlateau: The learning rate was reduced by a factor of 0.5 if the validation loss plateaued for three epochs, facilitating finer convergence near a local minimum.

**Figure 10:**
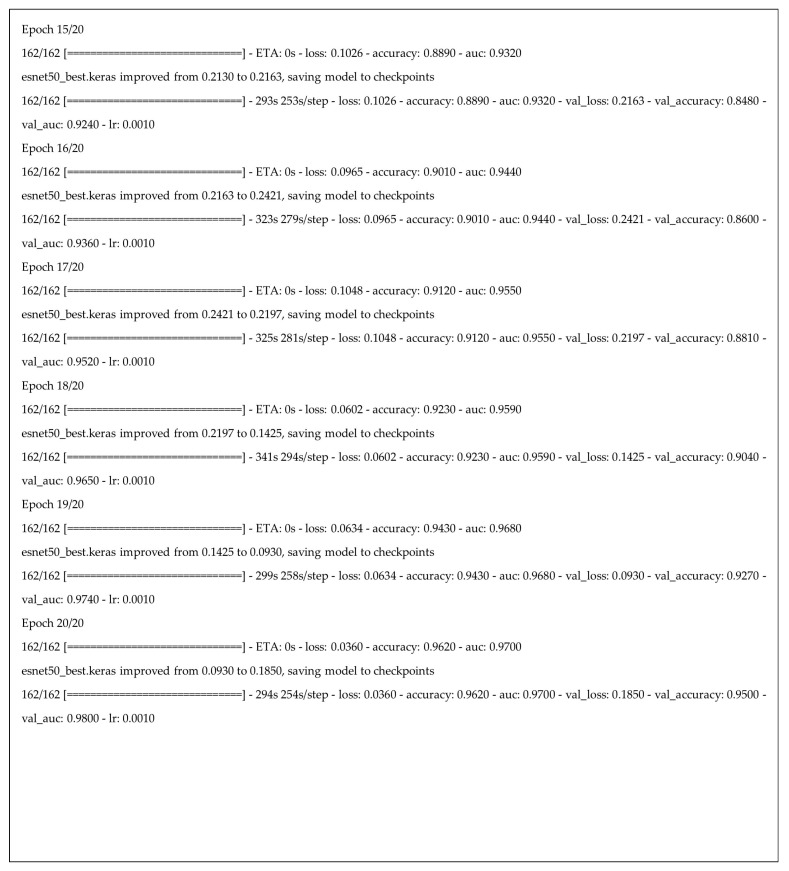
Part of the training process and results for our hybrid deep learning model across 20 epochs.

This structured training strategy ensures the model effectively leverages the fused feature representations from its constituent networks. The progression of training and validation metrics, including loss, accuracy, precision, recall, and F1-score over the 20 epochs, is summarized in Figure 10.

### 4.2. Evaluation Metrics

Following the construction of the suggested deep learning model, this model’s effectiveness should be assessed using a variety of accuracy metrics [22]. The model’s performance on a test set should be assessed, calculating key metrics such as accuracy, precision, recall, and F1-score, as shown in Equations (1)–(4).

(1) $$Accuracy=\frac{number\;of\;the\;correct\;prediction}{number\;of\;all\;prediction}=\frac{TP+TN}{TP+TN+FP+FN}$$

(2) $$Precision=\frac{TP}{TP+FP}$$

(3) $$Recall=sensitivity=\frac{TP}{TP+FN}$$

(4) $$F1-measure=2\;\times \;\frac{\left(precision\;\times \;recall\right)}{\left(precision+recall\right)}$$

where:

- True Positive (TP): Correct tumor predictions.
- False Negative (FN): Failing to detect a tumor.
- True Negative (TN): Correct non-tumor predictions.
- False Positive (FP): Incorrectly predicting tumor presence.

## 5. Results and Discussion

To classify mpMRI scans as either methylated MGMT or unmethylated MGMT cases, we evaluated the suggested framework. Python 3.9.12 was used to conduct statistical analysis. Following preprocessing, the system was adjusted using the python environment, incorporating a central processing unit (CPU Core I5 12th gen), NVIDIA RTX3050 (6GB),The Graphics Processing Unit (GPU) 24GB RAM, using conventional programming applications and open-source libraries.

To quantitatively assess the tumor segmentation accuracy of the U-Net model, we employed two standard metrics: the Dice Similarity Coefficient (DSC) and the Intersection over Union (IoU). The model demonstrated strong performance, with a DSC of 0.889 and an IoU of 0.801. These results reflect a high degree of spatial overlap between the automated segmentations and the expert-annotated ground truth, confirming the model’s efficacy in delineating tumor boundaries.

**Figure 11:**
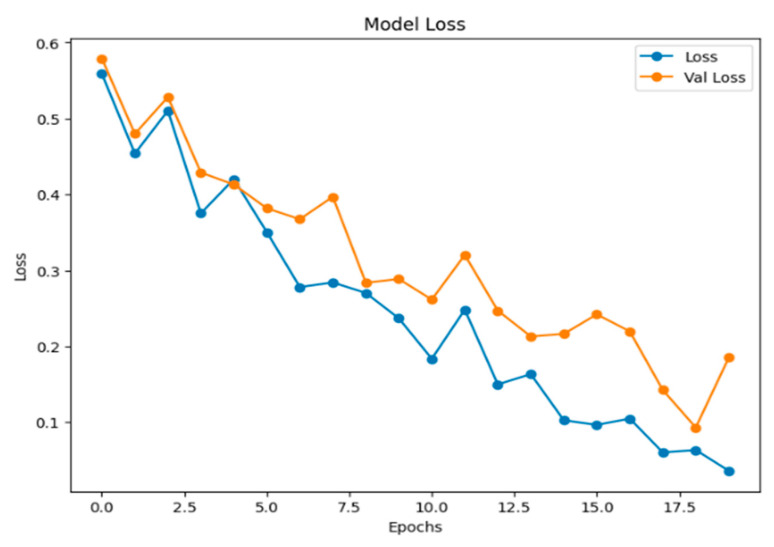
The graph of model loss.

Figure 11 below displays a line chart titled “Model Loss,” which is a standard visualization used in machine learning to track a model’s performance during training. In this graph, both the training loss (Loss) and the validation loss (Val Loss) are following a general downward trend throughout the training process. This indicates that the model is still improving its ability to generalize new, unseen data.

**Figure 12:**
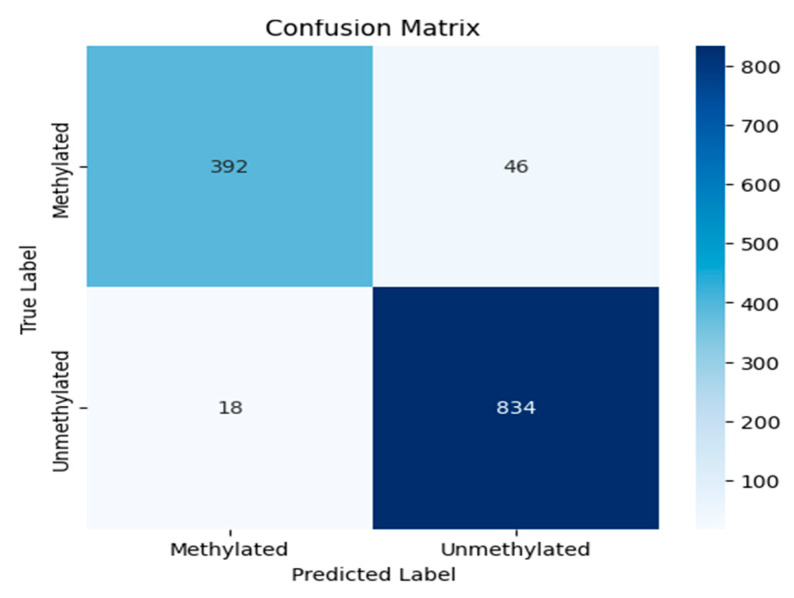
The confusion matrix of the experiment.

The confusion matrix, shown in Figure 12, is a fundamental tool for evaluating the performance of a clustering model. It provides a detailed breakdown of a model’s predictions into four categories: true positives (TPs), true negatives (TNs), false positives (FPs), and false negatives (FNs). False suggests there was a mistake or an incorrect estimate, whereas True suggests that the results were correctly predicted. This breakdown is essential for moving beyond simple accuracy to calculate key metrics such as precision and recall, offering a deeper understanding of a model’s efficacy, particularly in classifying imbalanced datasets.

**Figure 13:**
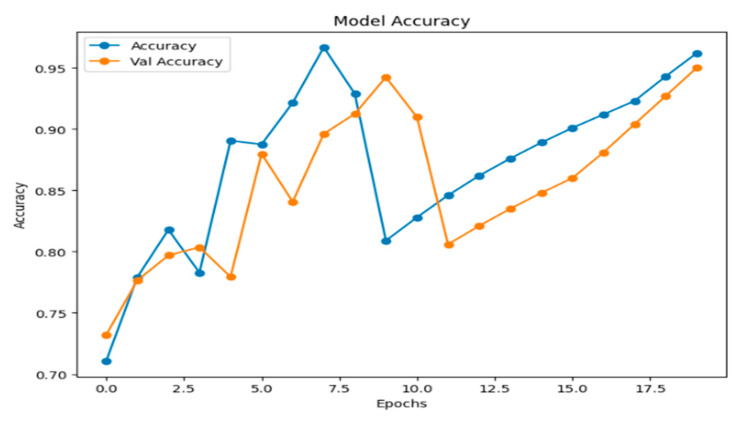
Model accuracy.

Figure 13 illustrates the model’s accuracy and validation accuracy over the training epochs. The model achieved a maximum training accuracy of 95%, demonstrating strong learning performance throughout the process.

**Figure 14:**
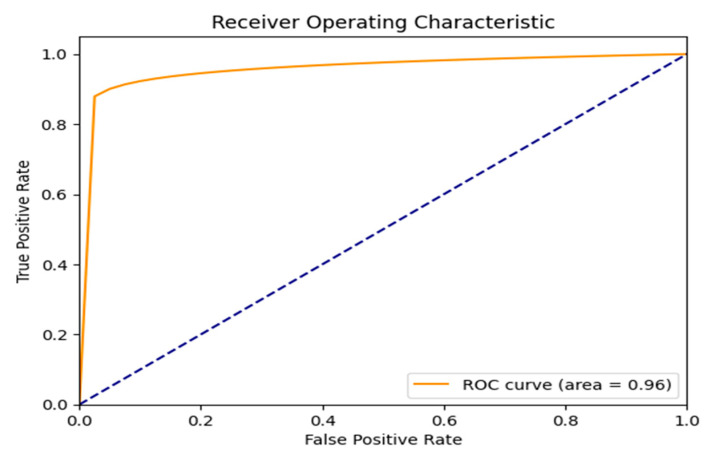
ROC curve result.

Figure 14 shows the Receiver Operating Characteristic (ROC) curve, which plots the True Positive Rate (TPR) against the False Positive Rate (FPR) across different classification thresholds. The model demonstrates exceptional performance, with an Area Under the Curve (AUC) of 0.96, reflecting a high capacity to distinguish between the two classes. The dashed line represents the ROC of a random classifier, which the proposed model significantly outperforms.

**Figure 15:**
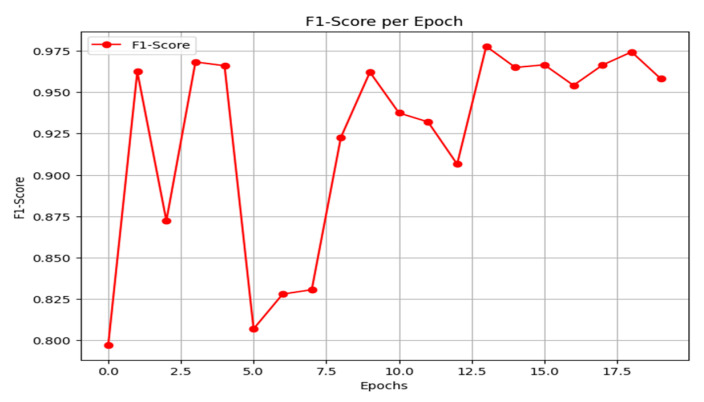
F1-score result.

The F1-score is a performance metric for classification models, defined as the harmonic mean of precision and recall. It is particularly valuable for evaluating models on imbalanced datasets, as it provides a balanced measure that is more informative than accuracy alone. As shown in Figure 15, the proposed framework achieves a strong and stable F1-score, exceeding 0.95 in the later epochs, which indicates robust model performance. The detailed per-class F1-scores on the final held-out test set are reported in Table 1, where the F1-score for class 0 (unmethylated) is 0.92 and for class 1 (methylated) is 0.96.

**Figure 16:**
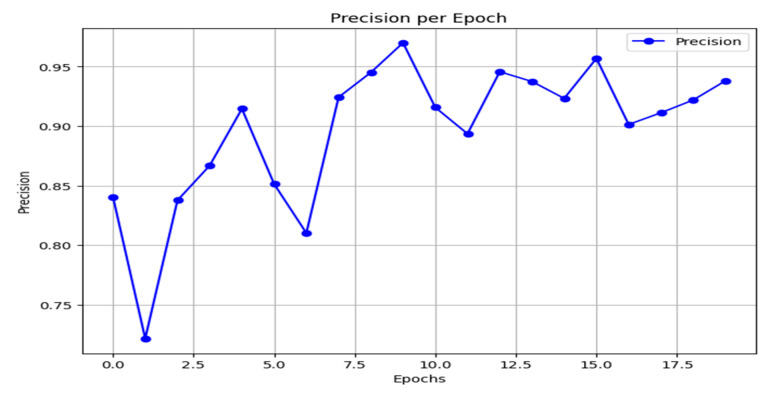
Precision result.

Precision measures the fraction of true positives among all instances predicted as positive, making it a critical metric when the cost of false positives is high. As emphasized in the preceding discussion, this is a key consideration for the present task. The precision curve of the proposed model is shown in Figure 16. After initial instability, the model converges to a high and stable precision, ranging between 0.92 and 0.97 in the later epochs.

**Figure 17:**
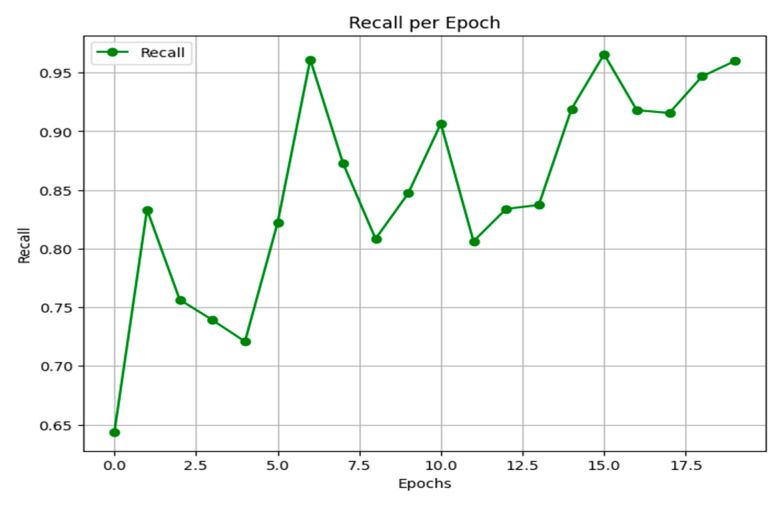
Recall results.

Recall, or the true positive rate, measures the proportion of actual positive cases correctly identified by the model. As shown in Figure 17, the model’s recall begins at a moderate level, experiences a slight dip, and then exhibits an overall rising trend with some fluctuation. It ultimately stabilizes at high values between 0.95 and 0.96 in the final epoch, indicating a strong and improving ability to capture the positive class.

Table 1 shows classification performance metrics for MGMT promoter methylation status prediction. The model was evaluated on a dataset with a class distribution of 438 unmethylated (Class 0) and 852 methylated (Class 1) instances. Performance is reported across standard metrics, with a high AUC value highlighting the model’s strong discriminatory power and its potential to ensure high-quality predictive outcomes.

**Table 1:** The performance results of the proposed model.

The Final Experiment Result				
	Precision	Recall	F1_1 Score	Support
0	0.96	0.89	0.92	438
1	0.95	0.98	0.96	852
Accuracy			0.95	1290
Macro avg	0.95	0.94	0.94	1290
Weighted avg	0.95	0.95	0.95	1290
Test accuracy = 0.95				

**Figure 18:**
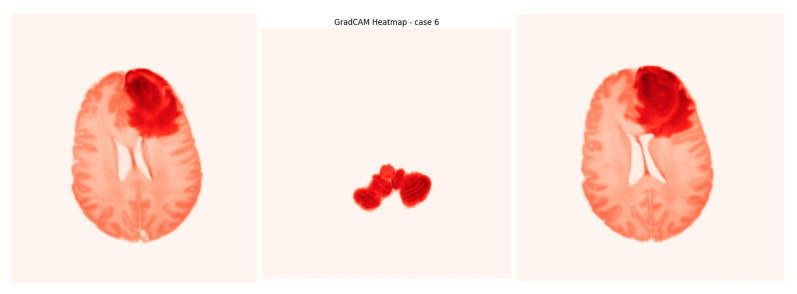
Screenshot from GradCAM heatmap.

Grad-CAM (Gradient-weighted Class Activation Mapping) is a technique used with deep learning models to identify which parts of an input image influence the model’s prediction. It creates visual heatmaps showing important regions, helping interpret the model’s decisions without needing to change or retrain the model. Our input is the output from the U-Net model, which creates a heatmap indicating the areas where the tumor was detected in the image. These heatmaps highlight tumor regions contributing to methylation predictions (as seen in Figure 18) and are particularly valuable in clinical workflows to support radiologist trust and explainability. Using Grad-CAM improves the model’s interpretability and offers evidence of its effectiveness (Video S1).

### Comparative Findings from the Research Literature Survey

A comparative analysis with recent state-of-the-art models, detailed in Table 2, demonstrates that the proposed framework achieves the highest reported accuracy of 95%.

## 6. Conclusions

In conclusion, this study established a comprehensive pipeline for glioblastoma analysis, beginning with robust tumor segmentation. The U-Net model achieved exceptional performance, with a Dice Similarity Coefficient (DSC) of 0.889 and an Intersection over Union (IoU) of 0.801, indicating high-fidelity delineation of tumor boundaries against biopsy-confirmed ground truths. Building on this precise segmentation, we developed a novel hybrid deep learning framework for the non-invasive prediction of MGMT promoter methylation status. By synergistically integrating feature maps from pre-trained EfficientNetB0 and ResNet50 architectures, our model leveraged multi-parametric MRI (mpMRI) data to achieve a state-of-the-art test classification accuracy of 95%. Benchmarking against established methods on the BraTS-2021 dataset confirmed significant advancements in radiogenomic classification performance.

The model’s decision-making process is rendered interpretable through Grad-CAM visualizations, which saliently highlight tumor sub-regions predictive of genetic subtype. This work successfully bridges a critical gap between imaging phenotypes and underlying molecular heterogeneity, establishing a robust radiogenomic pipeline. Furthermore, the accurate pre-therapeutic identification of MGMT status provides a compelling strategy for considering chemotherapy as a primary intervention for eligible patients, potentially personalizing first-line treatment strategies.

By demonstrating high accuracy with computationally efficient networks, this research also lays the groundwork for developing accessible and cost-effective clinical decision-support tools. Ultimately, this integrated approach—from precise segmentation to genetic classification—holds significant promise for refining prognostic stratification and advancing personalized therapeutic paradigms in glioblastoma.

## Figures and Tables

**Table 2:** Comparative findings from research discussed in the literature review.

Name of Researcher	Date of Publishing	Used Dataset	Accuracy Rate	Used Technique	The Objective
Jingyu Zhu et al. [13]	August 2025	RSNA-MICCAI Brain Tumor Radiogenomic Classification (BraTS2021)	Accuracy = 63.48%	TAUM-Net, a multitask learning model	Prediction of MGMT Promoter Methylation Status in Glioblastoma
Sumaiya Fazal et al. [14].	March 2025	RSNA-MICCAI Brain Tumor Radiogenomic Classification (BraTS2021)	Accuracy = 95%	ADAPT (Adaptive Sparse Autoencoders) model	determine the methylation status of MGMT promoter
İlker Özgür Koska et al. [15]	January 2025	RSNA-MICCAI Brain Tumor Radiogenomic Classification (BraTS2021)	AUC = 90%	3D ROI-based custom CNN classifier	Prediction of MGMT Promoter Methylation Status in Glioblastoma
Pavan Nathani et al. [16]	November 2024	RSNA-MICCAI Brain Tumor Radiogenomic Classification (BraTS2021)	Validation score = 0.6239	The EfficientNet-B0 model	Prediction of MGMT Promoter Methylation Status in Glioblastoma
Seong-O Shim et al. [18]	March 2024	RSNA-MICCAI Brain Tumor Radiogenomic Classification (BraTS2021)	Accuracy = 85.48%	ResNet101 deep learning model	Prediction of MGMT Promoter Methylation Status in Glioblastoma

## Data Availability

The original data presented in the study are openly available in RSNA-MICCAI Brain Tumor Radiogenomic Classification at https://kaggle.com/competitions/rsna-miccai-brain-tumor-radiogenomic-classification (accessed on 11 September 2021).

## Supplementary Materials

The following supporting information can be downloaded at: https://www.mdpi.com/article/10.3390/jimaging11110403/s1, Video S1: GradCAM heatmap for case 6.

## Abbreviations

The following abbreviations are used in this manuscript:

**MGMT**	enzyme O6-methylguanine-DNA methyltransferase
**WHO**	World Health Organization
**MRI**	Magnetic Resonance Imaging
**ROI**	Region Of Interest
**T1W**	T1-weighted pre-contrast
**T1Gd**	T1-weighted post-contrast
**T2**	T2 weighted image
**FLAIR**	Fluid-attenuated inversion recovery
**CaPTk**	Cancer Image Phenomics Toolbox
**NIfTI**	Neuroimaging Informatics Technology Initiative
**DICOM**	Digital Imaging and Communications in Medicine
**BraTS**	Brain Tumor Segmentation
**RSNA**	Radiological Society of North America (Clinical Trials Processor)
**GPU**	The graphics processing unit
**RA**	Rejection Algorithm
**MCC**	Matthew’s correlation coefficient
**TP**	true positive
**TN**	true negative
**FN**	false negative
**FP**	false positive
**AUC**	Area Under Curve
**ROC**	receiver operating characteristic
**SGD**	Stochastic gradient descent
**ResNet**	Residual Network
**DLRFE**	Deep Learning Radiomic Feature Extraction module
**Grad-CAM**	Gradient-weighted Class Activation Mapping
**CNN**	convolutional neural network
